# Does sleep-dependent consolidation favour weak memories?

**DOI:** 10.1016/j.cortex.2020.10.005

**Published:** 2021-01

**Authors:** Marit Petzka, Ian Charest, George M. Balanos, Bernhard P. Staresina

**Affiliations:** a School of Psychology and Centre for Human Brain Health, University of Birmingham, Birmingham, UK; b School of Sport, Exercise and Rehabilitation Sciences, University of Birmingham, Birmingham, UK

**Keywords:** Sleep, Episodic memory, Consolidation, Interference

## Abstract

Sleep stabilizes newly acquired memories, a process referred to as memory consolidation. According to recent studies, sleep-dependent consolidation processes might be deployed to different extents for different types of memories. In particular, weaker memories might benefit greater from post-learning sleep than stronger memories. However, under standard testing conditions, sleep-dependent consolidation effects for stronger memories might be obscured by ceiling effects. To test this possibility, we devised a new memory paradigm (Memory Arena) in which participants learned temporospatial arrangements of objects. Prior to a delay period spent either awake or asleep, training thresholds were controlled to yield relatively weak or relatively strong memories. After the delay period, retrieval difficulty was controlled via the presence or absence of a retroactive interference task. Under standard testing conditions (no interference), a sleep-dependent consolidation effect was indeed observed for weaker memories only. Critically though, with increased retrieval demands, sleep-dependent consolidation effects were seen for both weaker and stronger memories. These results suggest that all memories are consolidated during sleep, but that memories of different strengths require different testing conditions to unveil their benefit from post-learning sleep.

## 1 Introduction

How do fleeting experiences become long-term memories? Research has established the importance of post-learning sleep for the strengthening of recently acquired memories, a process referred to as memory consolidation (Diekelmann & Born, 2010; Jenkins & Dallenbach, 1924; Müller & Pilzecker, 1900; Bjoern Rasch & Born, 2013). However, the principles governing sleep-dependent consolidation, i.e., superior memory retention after sleep compared to wake, are still poorly understood. Does post-learning sleep benefit all memories equally, or are particular types of memories prioritized for consolidation processes? Consistent with the latter scenario, evidence has accumulated in recent years for a somewhat selective sleep-dependent consolidation process.

On the one hand, a greater benefit from post-learning sleep has been shown for emotionally salient compared to neutral stimuli (Hu, Stylos-Allan, & Walker, 2006), for events with high compared to low future relevance (Wilhelm et al., 2011) and for items intended to be later remembered compared to items intended to be forgotten (Rauchs et al., 2011; Saletin, Goldstein, & Walker, 2011). To the extent that emotional salience, high future relevance and the intention to remember entail deeper processing during encoding (Craik & Lockhart, 1972), these results suggest that sleep-dependent consolidation may prioritise stronger memories. Differential post-sleep memory outcomes might then result from a synaptic downregulation process during sleep through which weaker memories are pruned but stronger memories are preserved (Tononi & Cirelli, 2006).

On the other hand, there is evidence supporting the notion that sleep-dependent consolidation favours weaker memories. For instance, Bäuml, Holterman, and Abel (2014) compared sleep-dependent consolidation of items that were restudied with items that were retrieved during a practice period. As retrieval practice usually results in stronger memories than restudy ('testing effect', Roediger & Butler, 2011), their finding of restudied (i.e., weaker) and not retrieved (i.e., stronger) items showing a sleep-dependent consolidation effect suggests that weaker memories differentially benefit from sleep-dependent consolidation. Two other studies, also indirectly manipulating memory strength, came to the same conclusion. They have shown greater sleep-dependent consolidation effects for word pairs with low compared to high semantic relatedness (Lo, Dijk, & Groeger, 2014; Payne et al., 2012), where low semantic relatedness typically yields weaker memories. Moreover, experimentally facilitating consolidation during sleep via targeted memory reactivation (Rasch, Büchel, Gais, & Born, 2007; Rudoy, Voss, Westerberg, & Paller, 2009; Schreiner & Rasch, 2015) has been demonstrated to be more effective for items less well remembered prior to sleep (i.e., weaker memories) (Cairney, Lindsay, Sobczak, Paller, & Gaskell, 2016; Creery, Oudiette, Antony, & Paller, 2015). Finally, one study directly manipulated pre-sleep memory strength, either by having participants learn some stimuli to a lower criterion than others, or by imposing a retroactive interference task immediately after learning. Again, results indicate greater sleep-dependent consolidation benefits for weaker than for stronger memories (Drosopoulos, Schulze, Fischer, & Born, 2007).

How can these different lines of results be reconciled? One possible explanation for the result of weaker memories being preferentially consolidated during sleep is a ceiling effect for stronger memories. That is, elevating the strength of pre-sleep memory traces beyond a certain threshold might conceal the retention benefit typically afforded by sleep. In other words, sleep possibly benefits both weaker and stronger memories, but different testing protocols (mitigating ceiling effects) are needed to uncover these benefits. One effective means to reduce the impact of ceiling effects is to retroactively weaken memory traces through interference, thereby moving them away from ceiling. For instance, one study had participants learn word pairs to a 100% accuracy criterion (corresponding to rather strong pre-sleep memories) and applied retroactive interference immediately before the final (post-sleep) retrieval session (Ellenbogen, Hulbert, Stickgold, Dinges, & Thompson-Schill, 2006). This procedure indeed revealed a sleep-dependent consolidation effect, despite the initially high learning criterion. Critically though, that study did not vary pre-sleep memory strength, such that it is unclear whether sleep protects both weaker and stronger memories from retroactive interference.

In light of the extant findings, we hypothesised that both weaker and stronger memories might benefit from post-learning sleep, but that an increase in retrieval difficulty is needed to uncover sleep-dependent consolidation of stronger memories. To assess the beneficial effect of sleep-dependent consolidation on weaker and stronger memories as a function of retrieval difficulty within the same paradigm, we systematically manipulated (i) pre-sleep memory strength by varying a training threshold and (ii) retrieval difficulty by inducing retroactive interference. To this end, we devised a new memory paradigm (‘Memory Arena’), designed to capture de-novo learning of temporal and spatial aspects of episodic memory. Specifically, the Memory Arena paradigm has participants learn both the temporal and spatial position of 20 individual object images placed on a circle. Learning is in principle completed when all 20 objects are placed in the correct temporal order to their correct position (100% performance). Importantly though, memory strength can be experimentally controlled by terminating training at different performance levels. Retroactive interference was induced by having participants learn a new temporospatial arrangement of the same objects directly before the final retrieval.

Our first aim was to replicate the greater benefit of sleep-dependent consolidation for weaker relative to stronger memories (using a standard testing protocol without retroactive interference). Indeed, we found that weaker memories (training threshold of 1 × 50% accuracy) showed a sleep-dependent consolidation effect, whereas stronger memories (training threshold of 2 × 70% accuracy) did not. We then tested whether increased retrieval demands, i.e., the need to overcome retroactive interference, would yield a sleep-dependent consolidation effect for stronger memories as well. Intriguingly, this manipulation revealed sleep-dependent consolidation effects for both types of pre-sleep memories (weaker and stronger). These results suggest that post-learning sleep might benefit all memories, but that different testing conditions are differentially sensitive to unveiling consolidation of weaker versus stronger memories.

## 2 Method

We report how we determined our sample size, all data exclusions (if any), all inclusion/exclusion criteria, whether inclusion/exclusion criteria were established prior to data analysis, all manipulations, and all measures in the study.

### 2.1 Participants

Overall, 128 participants took part in the study. Eight participants were excluded – 6 participants based on Actigraph recordings (5 participants in a sleep group slept less than 5 h between pre- and post-retrieval and 1 participant in a wake group slept during the day), 1 participant did not finish the experiment and 1 participant was erroneously assigned to the wrong condition. The remaining 120 participants were included in the analyses (age = 20.58 ± 2.08 [mean ± SD], female = 83, n = 15 per group). Target sample size was based on two relevant studies using between-subjects designs. Drosopoulos et al. (2007) used 10 participants per group to demonstrate a greater sleep-dependent consolidation effect for weaker than for stronger memories. Ellenbogen et al. (2006) used 12 participants per group to show that sleep-dependent consolidation effects are impacted by retrieval demands.

Participants had no history of neurological or psychiatric disorders and had a normal sleep–wake cycle as assessed with a sleep diary. Inclusion/exclusion criteria were established prior to data analysis. For taking part in the study, participants received either monetary reimbursement or study credits. The study was approved by the University of Birmingham Research Ethics and Governance Committee and written informed consent was obtained from participants before the start of the experiment.

### 2.2 Task design & procedure

**Fig. 1:**
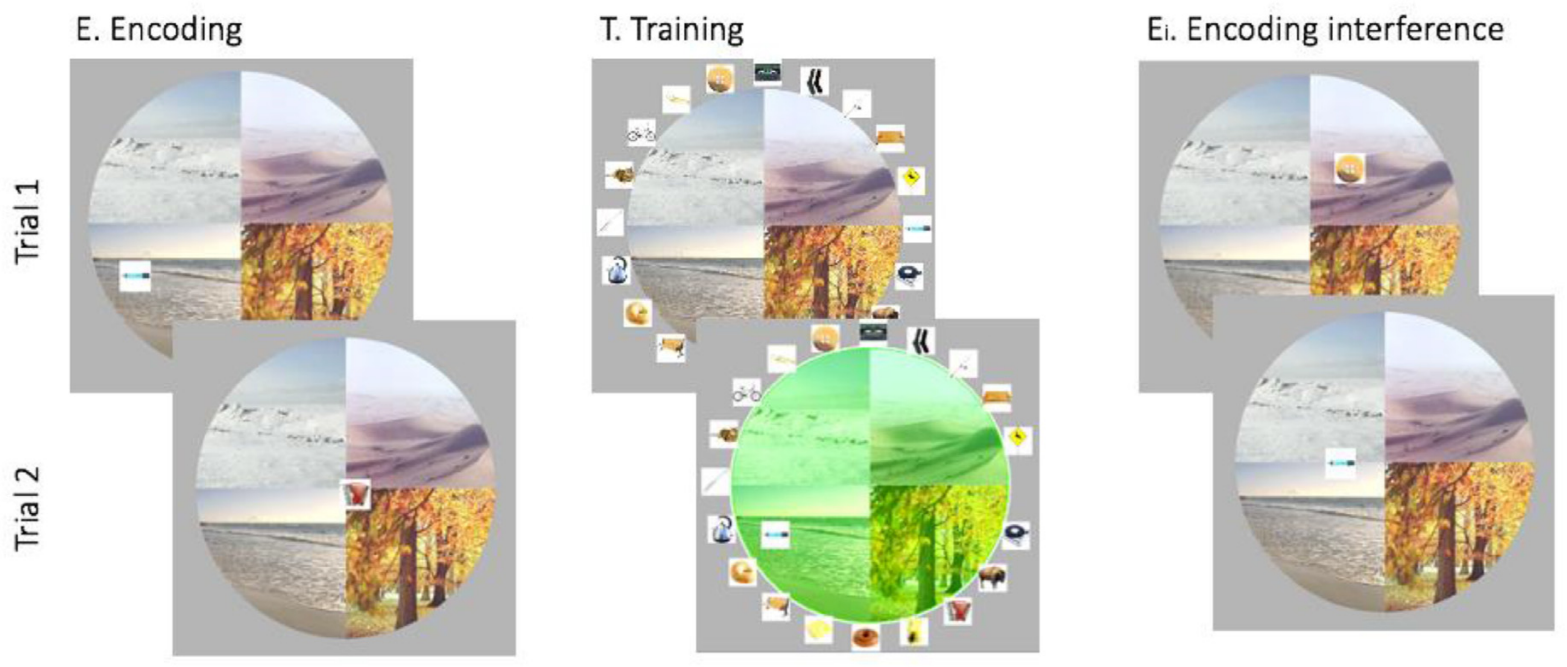
Task design. During encoding 20 objects were presented in the *Memory Arena*. After clicking on an object, the current object disappeared and the next object was shown. The training session started with all 20 objects randomly arranged around the arena. Objects had to be dragged and dropped in the correct sequence to the correct spatial position. Feedback was given after each trial and any errors were corrected. Interference was induced by encoding of the same objects but in a different sequence and at different spatial positions. Retrieval (not shown) followed the same procedure as training, but no feedback and correction were provided.

To capture both the temporal and spatial components of episodic memory we designed a new paradigm, called *Memory Arena*. It consists of a circle divided into four quarters, each depicting a different scene background (upper left: arctic landscape, upper right: desert, lower right: autumn forest, lower left: sea, Fig. 1). On top of these backgrounds, individual objects are presented in different spatial positions. Participants have to learn the temporal (sequential) and spatial position of each object.

Twenty target objects were randomly selected from a pool of 50 common animate and inanimate objects (Konkle, Brady, Alvarez, & Oliva, 2010, coloured and presented on a white 90x90 pixels square). The spatial position of each object was restricted by the outline of the *Memory Arena* and by the position of other objects. Thus, there was no overlap between objects but it was possible that an object covered multiple background scenes.

During the encoding part of the *Memory Arena*, all 20 objects were presented one after another and participants confirmed an object's spatial position by clicking on the object. The current object then disappeared and the next object was presented (Fig. 1). Participants were encouraged to associate the objects with each other and with the background scenes into a narrative.

A training session was introduced directly after the encoding part. The training started with all 20 objects randomly arranged around the arena. Participants had to drag and drop the objects in the correct sequence to the correct spatial position. If an error was made regarding the sequence or spatial position, the arena turned red and the error was corrected. If the object was placed at its correct sequential and spatial position, the arena turned green. The sequence position was scored as correct if object *i* was placed at the *i*th position. The spatial position of an object was scored as correct if the overlap between its position and the correct position was higher than 25%.

After feedback and potential error correction, the object remained at its correct spatial position and the next object had to be placed in the arena. When all 20 objects were placed, participants received feedback about their overall performance [(n correct objects/n of total objects), where an object was classified as correct when both the sequence and spatial position were correct].

To manipulate pre-sleep memory strength, participants finished training after meeting two different levels of performance. Pre-sleep memory strength was defined as ‘weaker’ when the performance criterion was set to 50%, reached in one training round (1 × 50%) and defined as ‘stronger’ when the performance criterion was set to 70%, reached in two consecutive training rounds (2 × 70%).

After finishing the training session, participants performed a pre-delay retrieval task. The retrieval started, like the training session, with all 20 objects randomly arranged around the arena and the objects had to be dragged and dropped in the correct sequence to the correct spatial position. Importantly though, no feedback was provided, and errors were not corrected meaning that the objects remained at the spatial position where they were dropped.

As we compared memory performance between two retrieval tasks performed at different times of day (pre *vs* post-delay retrieval, AM *vs* PM or PM *vs* AM), an alternative explanation for a change in memory performance might be a change in attention/level of alertness. We thus employed a psychomotor vigilance task (PVT) directly before both retrieval tasks. The PVT started with a white fixation cross presented in the middle of the screen. After an average of 6 sec (with a jitter of ± 4 sec), the fixation cross was replaced by a counter starting at 0 and counting forwards to 2000 in 20 msec steps. During that time, participants had to press the space bar as fast as possible. Feedback about their reaction time was provided after the key press (displayed for 2sec).

In session one encoding, training, PVT and pre-delay retrieval were completed. 12 h later participants returned to the lab for the second session. For half of the participants the second session started with an interference task, designed to increase the post-delay retrieval difficulty. Participants were not informed about the interference task until the beginning of the second session. During the interference task, participants were asked to encode the same objects presented in a different sequence and at different spatial positions (Fig. 1). The new spatial position of every object was more than 5 pixels away from its original spatial position (centre to centre distance). Encoding of the interfering temporospatial arrangement was implemented in the same way as the original encoding. Following encoding, participants performed a retrieval session of the interfering arrangement (no training was conducted for the new arrangement). Finally, participants performed a second PVT and the retrieval task for the original arrangement (post-delay retrieval). All participants in the no-interference condition directly started with the PVT and the post-delay retrieval of the original arrangement.

### 2.3 Study design

**Fig. 2:**
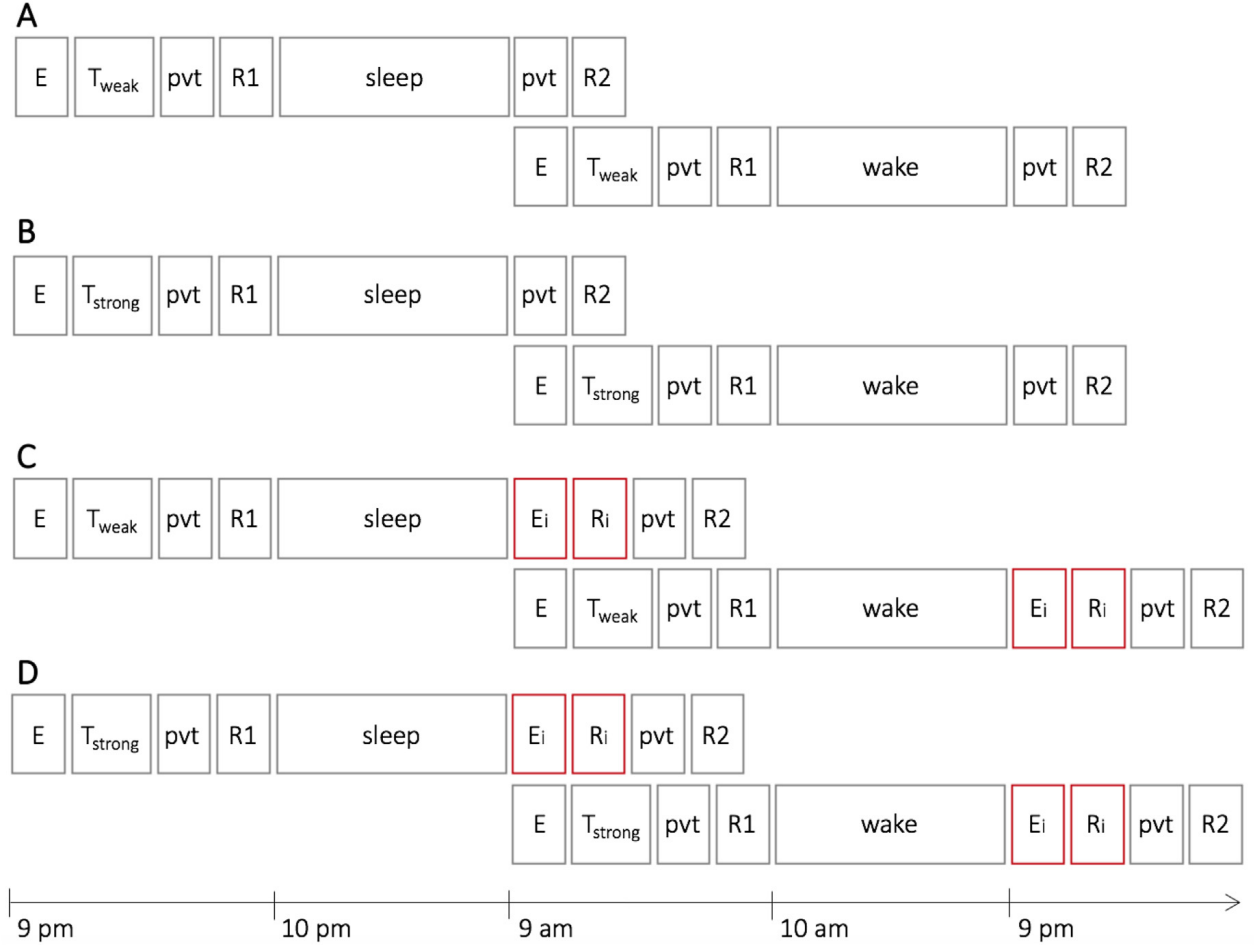
Study design. 120 participants were randomly assigned to one of 8 conditions (groups). All *sleep* groups performed the encoding, training and pre-delay retrieval in the evening and the post-delay retrieval 12 h later in the morning. The *wake* groups performed the encoding, training and pre-delay retrieval in the morning and the post-delay retrieval 12 h later in the evening. Besides the between-subjects factor Delay (sleep *vs* wake), pre-sleep memory strength was manipulated via the training threshold (A and C for weaker memories and B and D for stronger memories). Additionally, half of the participants were given an interference task before the post-delay retrieval to increase retrieval difficulty (between-subjects factor Retrieval Difficulty, A and B for no interference, C and D for interference). E = encoding, T = training, pvt = Psychomotor Vigilance Task, R1 = pre-delay retrieval, R2 = post-delay retrieval, i = interference.

We used a 2 (Delay: sleep *vs* wake) x 2 (Memory Strength: weaker *vs* stronger) x 2 (Retrieval difficulty: no interference *vs* interference) between-subjects design and participants were randomly assigned to one of the resulting 8 conditions (Fig. 2).

Participants in the sleep conditions performed the first session including encoding, training, PVT and pre-delay retrieval in the evening around 9 pm. After finishing the first session they went home to sleep. 12 h later, at 9 am, they returned to the lab to perform the second session. Half of the participants additionally conducted the interference at the beginning of the second session while the other half directly started with the PVT and post-delay retrieval. Participants in the wake conditions followed the same protocol shifted by 12 h, i.e., performing the first session (encoding, training, PVT and pre-delay retrieval) at 9 am and returning to the lab 12 h later at 9 pm for the second session (interference, PVT and post-delay retrieval or PVT and post-delay retrieval, respectively).

### 2.4 Data collection & analysis

The *Memory Arena* was implemented with MATLAB 2016a (MathWorks). Behavioural responses were recorded using the mouse. Data were prepared and analysed using MATLAB and statistical analyses were conducted with the statistical software R. For data visualization raincloud tools in R were used (Allen, Poggiali, Whitaker, Marshall, & Kievit, 2019; van Langen, 2020).

To capture memory performance, we considered two variables: sequence performance and spatial error (placement distance). Sequence performance was based on correct transitions within the sequence (3rd object is chosen after the 2nd object) rather than on the absolute sequential position (3rd object is chosen at 3rd position), as the absolute sequential position is not necessarily the most sensitive measurement for memory performance. For example, if the second object in the sequence was erroneously placed first but then the order was correctly remembered for all subsequent objects, scoring the absolute positions would yield a performance score of 0. However, by scoring the transition between objects, all but the last (which now comes after the 19th placement but should have come first) are correct. Hence, the sequence performance was calculated based on the difference between object x_i_ and object x_i-1_, where _i_ is the selected sequence position of object x. If the transition is correct this difference is 1. The sum of the correct transitions was then divided by the total number of possible transitions (n = 19 when n_obj_ = 20) and multiplied with 100 to get a percentage score. The spatial error was calculated using the Euclidean distance (in pixels) between the centre of the original position and the centre of the placed position of every object.

To test the effects of our experimental factors (Fig. 2), parametric ANOVAs were applied. Welch's *t*-tests were used as post-hoc comparisons as variances between groups were not always equal. Note that for Welch's *t*-tests, degrees of freedom are adjusted according to the Welch–Satterthwaite equation. For effect sizes, we report partial eta squared (η_p_^2^) for ANOVAs and Cohens d for Welch's *t*-tests. Shapiro–Wilk tests were applied to test for normal distributions of pre- and post-delay performance.

As traditional null-hypothesis testing does not allow for conclusions about the absence of an effect, we also conducted Bayesian analyses for all non-significant effects using the BayesFactor package in R (Morey & Rouder, 2015). According to the BayesFactor package, we used a Cauchy distribution (0, .707) as a prior. The Bayes factor BF_01_ (BF_01_ = 1/BF_10_) informs about the likelihood to observe the data if the null hypothesis is true [P (D |H_0_)/P (D| H_1_)]. A Bayes factor (BF_01_) between 1 and 3 can be considered as anecdotal evidence, 3–10 as moderate, 10–30 as strong, 30–100 as very strong and >100 as extreme evidence for H_0_ (Lee & Wagenmakers, 2013)_._

No part of the study procedures and analyses was pre-registered prior to the research being conducted.

## 3 Results

### 3.1 General

Our study design included the between-subjects factors Delay (sleep *vs* wake), Memory Strength (weaker *vs* stronger) and Retrieval Difficulty (no interference *vs* interference) resulting in 8 conditions with 15 participants each (Fig. 2).

First, we assessed whether the encoding strength manipulation (1 × 50% *vs* 2 × 70% training performance) affected the training duration, quantified both in terms of training rounds required and total time spent to reach criterion, including the encoding part (see Supplemental Information, Table S1 for descriptive data). We conducted a 2 × 2 × 2 ANOVA with the between-subjects factors Delay, Memory Strength and Retrieval Difficulty and the training duration or the total number of training rounds as dependent variables. For strong memories (2 × 70% criterion), participants required significantly longer training time [main effect of Memory Strength: F (1, 112) = 8.05, *p* = .005, η_p_^2^ = .07, mean_2x70%_ = 1128.13 sec, 95%CI_2x70%_ = 92.24 sec, mean_1x50%_ = 909.85 sec, 95%CI_1x50%_ = 120.61 sec] and more training rounds [main effect of Memory Strength: F (1, 112) = 15.58, *p* < .001, η_p_^2^ = .12, mean_2x70%_ = 4.88, 95%CI_2x70%_ = .49, mean_1x50%_ = 3.22, 95%CI_1x50%_ = .67]. The time of the day for training [evening for all sleep groups and morning for all wake groups) neither impacted training duration (main effect of Delay: F (1, 112) = .03, *p* = .869, η_p_^2^< .01, BF_01_ = 5.08] nor the number of training rounds needed to reach the criterion [main effect of Delay: F (1, 112) = 1.40, *p* = .239, η_p_^2^ = .01, BF_01_ = 2.89]. Likewise, there was no significant difference in training duration and number of training rounds between no interference and interference groups [duration: main effect of Retrieval Difficulty: F (1, 112) = .02, *p* = .884, η_p_^2^< .01, BF_01_ = 5.10; rounds: main effect of Retrieval Difficulty: F (1, 112) = .01, *p* = .937, η_p_^2^< .01, BF_01_ = 5.13].

Next, we identified one performance metric for all subsequent analyses. The *Memory Arena* paradigm yields two separate measures for memory performance, i.e., sequence memory and spatial memory. This allowed us to proceed with the memory measure most sensitive to our critical encoding strength manipulation (1 × 50% *vs* 2 × 70%, pre-delay Memory Strength). At the same time, we wanted to ensure that pre-delay memory performance did not differ between sleep and wake groups (factor Delay) or between no interference and interference groups (factor Retrieval Difficulty). We thus compared sequence performance as well as spatial error at pre-delay retrieval in two separate 2 × 2 × 2 ANOVAs, each including the between-subjects factors Delay, Memory Strength and Retrieval Difficulty. As expected, the 2 × 70% training threshold led to better pre-delay retrieval performance than the 1 × 50% training threshold for both measures [main effect of Memory Strength for sequence performance: F (1, 112) = 73.44, *p* < .001, η_p_^2^ = .40; main effect of Memory Strength for spatial error: F (1, 112) = 46.80, *p* < .001, η_p_^2^ = .29]. Critically though, the corresponding effect size was markedly higher for sequence (η_p_^2^ = .40) than for spatial memory performance (η_p_^2^ = .29). Consequently, we focused our subsequent analyses on sequence performance (but see Supplemental Information, Table S2 and Figure S1-S3, for analyses using spatial memory performance and Table S3 and Figure S4-S6 for analyses using overall memory performance). Importantly, neither Delay (sleep *vs* wake) nor Retrieval Difficulty (no interference *vs* interference) had a significant effect on sequence or spatial memory performance at pre-delay retrieval (all F < 1.29, all p > .258, all BF_01_ > 3.36), ensuring there were no other baseline (pre-delay) differences between groups.

To account for potential differences in attention between pre- and post-delay retrieval, we compared the number of attention lapses (reaction times > 500 msec, Basner & Dinges, 2011) during the psychomotor vigilance task (PVT). Results showed that there was no significant change in the number of lapses in any of the conditions from pre to post-delay retrieval (all t < 1.59, all p > .135), ruling out fatigue as a confounding factor for our results.

Consolidation, i.e., the change in sequence memory performance from pre to post-delay retrieval, was calculated as a relative change. In the following, sequence consolidation denotes the performance during post-delay retrieval relative to pre-delay retrieval, meaning that values > 100% reflect an increase, values < 100% reflect a decrease and values = 100% reflect a stabilization of sequence memory performance. Sleep-dependent consolidation is then defined as the differential consolidation effect for the sleep group compared to the corresponding wake group (factor Delay).

As a first analysis we conducted a 2 × 2 × 2 ANOVA with sequence consolidation as the dependent variable and Delay (sleep *vs* wake), Memory Strength (weaker *vs* stronger) and Retrieval Difficulty (no interference *vs* interference) as between-subjects factors. Across all groups, post-relative to pre-delay performance was higher in sleep groups than in wake groups [main effect for Delay: F (1,112) = 32.69, *p* < .001, η_p_^2^ = .23] and lower for high retrieval difficulty in comparison to low retrieval difficulty [main effect for Retrieval Difficulty: F (1,112) = 44.07, *p* < .001, η_p_^2^ = .28]. Neither the main effect for Memory Strength nor any of the two way interactions reached significance (all F < 2.06 all p > .154, all BF_01_ > 2.95). Critically though, we found a significant three way interaction [F (1, 112) = 6.21, *p* = .014, η_p_^2^ = .05], suggesting that sleep-dependent consolidation effects for weaker and stronger memories might differ as a function of retrieval difficulty. We thus conducted two sets of subsidiary ANOVAs: First, breaking up the factor Retrieval Difficulty, we conducted separate ANOVAs to test for sleep-dependent consolidation effects for weaker versus stronger memories under standard testing conditions (no interference, see section 3.2.) and with an increase in retrieval difficulty (interference, see section 3.3.). Second, breaking up the factor Memory Strength, we conducted separate ANOVAs to assess sleep-dependent consolidation effects as a function of retrieval difficulty for weaker and stronger memories, respectively (see section 3.4.).

### 3.2 No interference: only weaker memories show a sleep-dependent consolidation effect

**Fig. 3:**
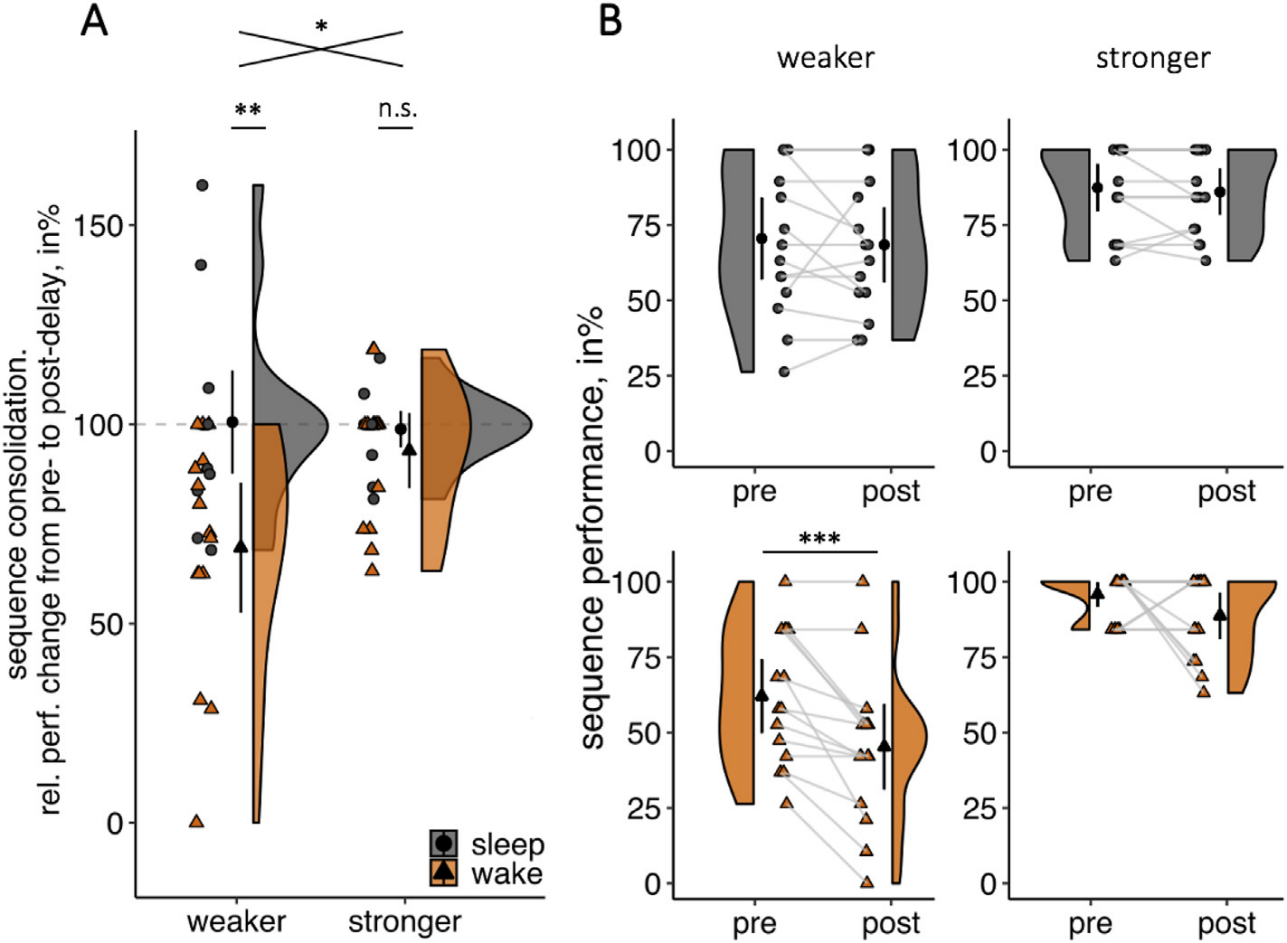
Consolidation effects for *no interference* groups. **A.** For weaker memories, sequence consolidation (relative performance change from pre-to post-delay retrieval) is significantly greater in the sleep group (grey, circle) than in the wake group (orange, triangle), whereas there is no statistical difference between the sleep and wake group for stronger memories. **B.** For stronger memories (*right column*), pre-as well as post-delay sequence performance was at ceiling, while pre- and post-delay sequence performance for weaker memories (*left column*) were normally distributed. Only sequence performance for weaker memories in the wake group significantly decreased from pre-to post-delay retrieval. Single participant data (grey filled circles for sleep groups and orange filled triangles for wake groups), density plots and group means with 95% CIs are shown in A and B. ∗ = *p* ≤ .05; ∗∗ = *p* < .01; ∗∗∗ = *p* < .001; n.s. = not significant, *p* > .1.

To test whether weaker memories show a greater sleep-dependent consolidation effect than stronger memories under standard testing conditions, a 2x2 ANOVA with the between-subjects factors Delay (sleep *vs* wake) and Memory Strength (weaker *vs* stronger) was conducted on sequence consolidation for the *no interference* groups. Overall, sequence consolidation was significantly greater in the sleep groups than in the wake groups [main effect of Delay: F (1,56) = 11.59, *p* = .001, η_p_^2^ = .17]. Interestingly though, this sleep-dependent consolidation effect was modulated by the initial memory strength [interaction of Delay x Strength: F (1,56) = 5.78, *p* = .020, η_p_^2^ = .09]. Post hoc *t*-tests revealed that sequence consolidation did not significantly differ between the sleep and the wake group for stronger memories [t (20.40) = 1.11, *p* = .28, d = .41, BF_01_ = 1.82]. However, for weaker memories, the sleep group showed significantly greater sequence consolidation than the wake group [t (26.63) = 3.25, *p* = .003, d = 1.19, Fig. 3A]. These results are consistent with the notion that sleep-dependent consolidation selectively benefits weaker memories.

As mentioned in the introduction, beneficial effects of sleep for weaker and not for stronger memories might result from stronger memories being at ceiling. Indeed, the distribution of pre-delay sequence performance for stronger memories significantly deviated from a normal distribution (assessed via Shapiro–Wilk tests) for the wake (W = .56, *p* < .001) and the sleep group (W = .80, *p* = .004) and was skewed towards high performance values (Fig. 3B). Conversely, the distribution of pre-delay sequence performance for weaker memories did not significantly differ from a normal distribution (wake group: W = .95, *p* = .513, sleep group: W = .92, *p* = .229). Under normal testing conditions (in the absence of retroactive interference), post-delay sequence performance for stronger memories was still at ceiling for both the wake (W = .78, *p* = .002) and the sleep group (W = .85, *p* = .018), thus likely to obscure any benefit of sleep for the consolidation of stronger memories (see Supplemental Information, Table S5 for Shapiro–Wilk tests of all distributions and Table S4 for *t*-tests of pre-versus post-delay sequence performance).

### 3.3 Interference: stronger memories also show a sleep-dependent consolidation effect

As mentioned above, stronger memories may require an increase in retrieval difficulty to mitigate possible ceiling effects and to unveil the beneficial effect of post-learning sleep for consolidation. To test this hypothesis, we again compared sequence consolidation in a 2x2 ANOVA with the between-subjects factors Delay (sleep *vs* wake) and Memory Strength (weaker *vs* stronger), this time focusing on the high retrieval difficulty (interference) groups.

**Fig. 4:**
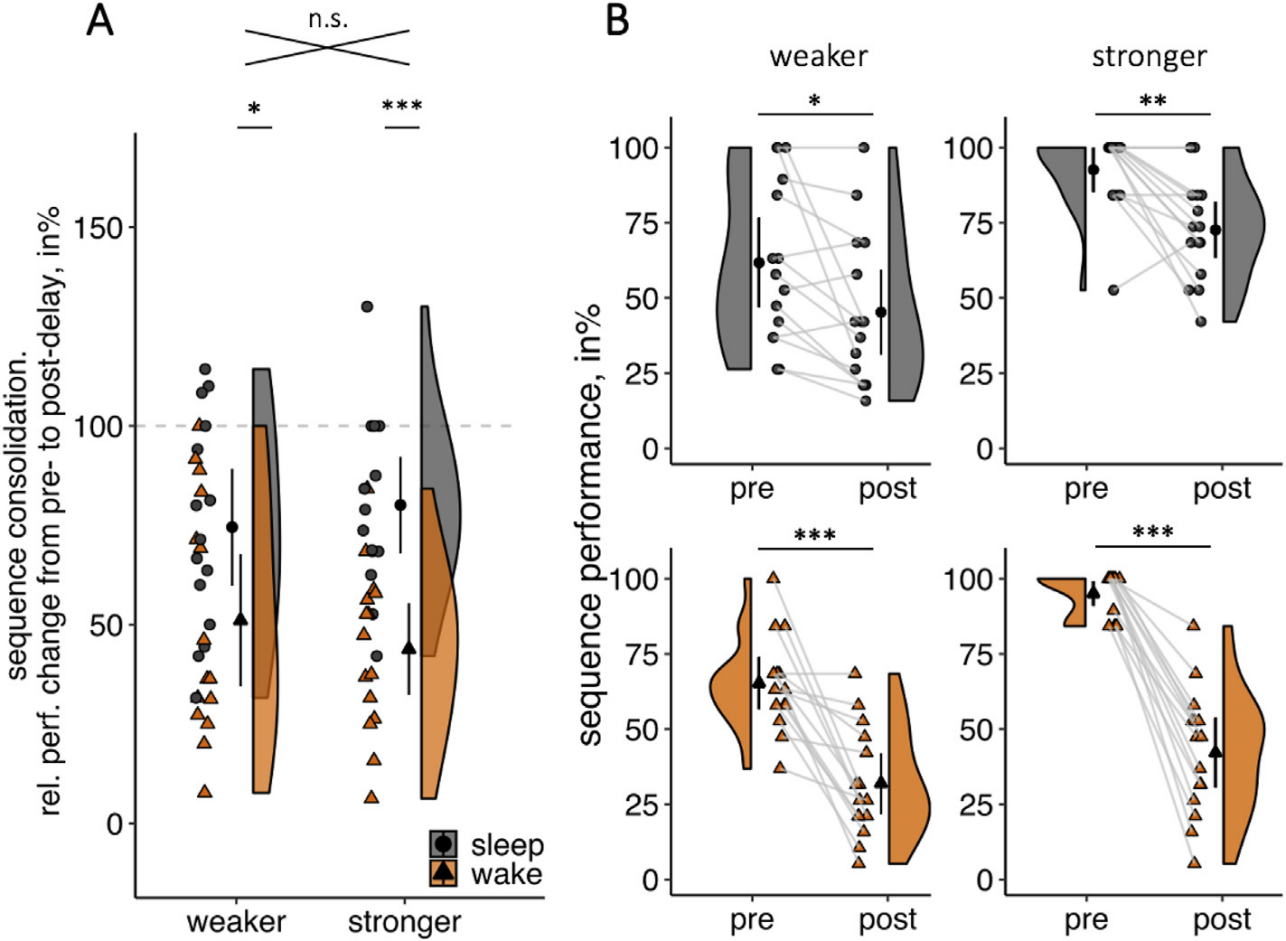
Consolidation effects for *interference* groups. **A.** After inducing retroactive interference, sequence consolidation (relative performance change from pre-to post-delay retrieval) in the sleep group (grey, circle) is significantly greater for both weaker and stronger memories. **B.** For stronger memories (*right column*), pre-delay sequence performance was at ceiling, while post-delay sequence performance was normally distributed. For weaker memories (*left column*), pre-as well as post-delay sequence performance were normally distributed. Sequence performance of all memories significantly decreased from pre-to post-delay retrieval. Single participant data (grey filled circles for sleep groups and orange filled triangles for wake groups), density plots and group means with 95% CIs are shown in A and B. ∗ = *p* ≤ .05; ∗∗ = *p* < .01; ∗∗∗ = *p* < .001; n.s. = not significant, *p* > .1.

Results demonstrated that sequence consolidation in the sleep groups was again significantly higher than in the wake groups [main effect of Delay: F (1,56) = 21.22, *p* < .001, η_p_^2^ = .28]. Importantly though, both weaker and stronger memories showed a significant sleep-dependent consolidation effect [no Delay × Strength interaction: F (1,56) = .98, *p* = .327, η_p_^2^ = .02, BF_01_ = 2.80]. For weaker memories, sequence consolidation was significantly greater in the sleep group than in the wake group [t (27.61) = 2.27, *p* = .031, d = .83], which replicated the pattern observed with low retrieval difficulty (see above). Critically though and in contrast to the low retrieval difficulty conditions, sequence consolidation was also significantly greater in the sleep group than in the wake group for stronger memories [t (27.93) = 4.64, *p* < .001, d = 1.70, Fig. 4A]. Indeed, the increase in retrieval difficulty effectively eliminated ceiling effects during post-delay retrieval (sleep group: W = .96, *p* = .722, wake group: W = .98, *p* = .981, Fig. 4B). These findings indicate that both stronger and weaker memories benefited from post-learning sleep, but that stronger memories required additional retrieval demands to show a benefit from post-learning sleep.

### 3.4 Sleep-dependent consolidation of stronger memories is modulated by retrieval difficulty

**Fig. 5:**
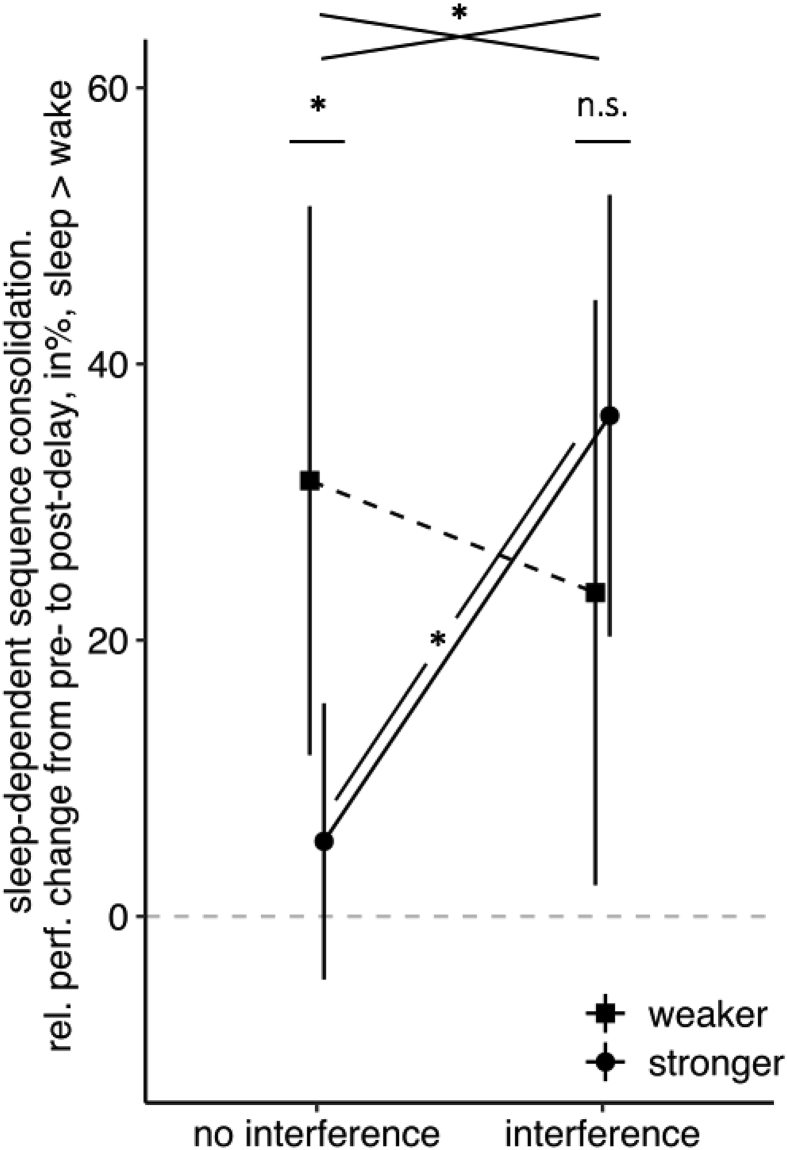
Sleep-dependent consolidation effects. With low retrieval difficulty (no interference, *left*), the difference in sequence consolidation (relative performance change from pre-to post-delay retrieval) between sleep and wake is significant for weaker memories only. With an increase in retrieval difficulty (interference, *right*), sleep-dependent consolidation effects are seen for both weaker and stronger memories. For stronger memories, the sleep-dependent consolidation effect was significantly greater for high retrieval difficulty (retroactive interference) compared to low retrieval difficulty (no interference). Shown are differences in means between sleep and wake groups and the corresponding 95% CIs. ∗ = *p* ≤ .05; n.s. = not significant, *p* > .1.

Our previous analyses showed a sleep-dependent consolidation effect for stronger memories when retrieval difficulty was increased (interference; see section 3.3.), but not under standard testing conditions (no interference; see section 3.2.). To directly test for changes in sleep-dependent consolidation effects from low to high retrieval difficulty separately for weaker and stronger memories, two 2x2 ANOVAs with the between-subjects factors Delay (sleep *vs* wake) and Retrieval Difficulty (no interference *vs* interference) were performed. For weaker memories, the increase in retrieval difficulty did not affect sleep-dependent consolidation effects [no interaction Delay x Retrieval Difficulty: F (1,56) = .33, *p* = .570, η_p_^2^ = .01, BF_01_ = 3.45]. For stronger memories, we found a significant increase in sleep-dependent consolidation effects from low to high retrieval difficulty [interaction of Delay x Retrieval Difficulty: F (1,56) = 11.21, *p* = .001, η_p_^2^ = .17, Fig. 5].

## 4 Discussion

The aim of the present study was to assess whether sleep-dependent memory consolidation favours weaker over stronger memories. To this end, we devised a novel memory paradigm (*Memory Arena*, Fig. 1) and experimentally controlled delay type (sleep or wake), pre-delay memory strength (weaker or stronger) and retrieval difficulty (no interference or interference) (Fig. 2). Under standard retrieval conditions (no retroactive interference), our data indeed suggest that weaker memories benefit from sleep while stronger memories seem not to (Fig. 3). This finding is in agreement with a growing body of evidence for sleep-dependent consolidation processes favouring weaker memories. Some of these studies used rather indirect manipulations of memory strength, e.g., by comparing retrieval versus restudy (Bäuml et al., 2014), by varying the difficulty of motor sequences from low element sequences (resulting in stronger procedural memories) to high element sequences (resulting in weaker procedural memories, Kuriyama, Stickgold, & Walker, 2013) or by changing the difficulty of a problem solving task (Sio, Monaghan, & Ormerod, 2013). Other studies directly manipulated memory strength either by varying the number of presentations (Denis, Schapiro, et al., 2020; Drosopoulos et al., 2007; Schapiro et al., 2017; Sheth, Varghese, & Truong, 2012), by inducing retroactive interference immediately after encoding to weaken memories (Drosopoulos et al., 2007; McDevitt, Duggan, & Mednick, 2015) or by comparing participants with high versus low pre-sleep memory performance (Diekelmann, Born, & Wagner, 2010; Djonlagic et al., 2009).

One factor potentially accounting for diminished sleep-dependent consolidation effects for stronger memories is that memory strength is often manipulated by repeated encoding and retrieval of the study material (Denis, Schapiro, et al., 2020; Drosopoulos et al., 2007; Schapiro et al., 2017; Sheth et al., 2012). That is, it has been argued that online retrieval emulates a consolidation process similar to that occurring during sleep (Antony, Ferreira, Norman, & Wimber, 2017). Consequently, stronger memories might already be sufficiently consolidated before sleep, yielding less need for further consolidation during sleep. Convergent evidence for this notion comes from studies examining post-learning sleep spindle activity, with spindles being considered a key mechanistic vehicle of memory consolidation (Fernandez & Lüthi, 2020; Peyrache & Seibt, 2020). In particular, an increase in spindle power during a post-learning nap has been reported after a high difficulty learning task (producing weaker memories) but not after a low difficulty learning task (producing stronger memories) (Schmidt et al., 2006). Likewise, spindle density has been linked to consolidation specifically of weaker memories (Denis, Mylonas, et al., 2020). Two other nap studies used targeted memory reactivation (TMR) to experimentally bolster consolidation. Interestingly, TMR resulted in better post-sleep memory performance only for weakly encoded memories (Cairney et al., 2016; Creery et al., 2015). Collectively, these findings suggest that sleep-dependent consolidation processes are preferentially deployed for weaker compared to stronger memories.

However, one alternative explanation – at least for the behavioural effects described above – is that beneficial effects of sleep for stronger memories are obscured by ceiling effects. In the present study, we demonstrate that under normal retrieval conditions (without interference), ceiling effects for stronger memories during pre-delay retrieval still persist during post-delay retrieval and would thereby conceal possible sleep-dependent consolidation effects. One way to eliminate ceiling effects during post-delay retrieval is to induce retroactive interference directly before retrieval. This approach has been taken in a series of studies explicitly testing the protective effect of sleep against retroactive interference. Indeed, despite training participants to 100% pre-delay memory accuracy, the introduction of retroactive interference after the delay and before the final retrieval revealed a beneficial effect of sleep over wake, i.e., a sleep-dependent consolidation effect (Ellenbogen, Hulbert, Jiang, & Stickgold, 2009; Ellenbogen et al., 2006; but see; Bailes, Caldwell, Wamsley, & Tucker, 2020; Pöhlchen, Pawlizki, Gais, & Schönauer, 2020). In line with these studies, we used retroactive interference to increase retrieval difficulty and thereby push memory performance from ceiling. Critically, this manipulation revealed sleep-dependent consolidation effects for weaker as well as for stronger memories (Fig. 4). One interesting question for future research is whether this ‘rescue’ of sleep-dependent consolidation effects for strong memories relies on interference manipulations, or whether other means of increasing retrieval demands, e.g., dual task manipulations, produce similar effects.

Our current results thus suggest that post-learning sleep benefits all memories, but that greater levels of initial memory strength call for adjusted testing protocols. It is interesting to note that weaker memories benefitted from sleep irrespective of subsequent retrieval demands, at least with respect to the presence versus absence of retroactive interference as employed here. That said, an important goal for future research is to establish the lower memory strength boundaries for sleep-dependent consolidation effects to occur. In particular, if initial memory strength is too low, a floor effect would likely hinder any benefit from subsequent sleep.

It deserves mention that besides retrieval difficulty, a number of other factors appear to impact sleep-dependent consolidation. One such factor is the duration of sleep. While some studies used 2 h daytime naps as a delay period (Cairney et al., 2016; Creery et al., 2015; Denis, Mylonas, et al., 2020; Schmidt et al., 2006), others followed a whole-night protocol (Bäuml et al., 2014; Diekelmann et al., 2010; Drosopoulos et al., 2007). Importantly, Schapiro et al. (2017) demonstrated that a full night of sleep and a nap show differential selectivity for weaker or stronger memories. In line with other nap studies (Cairney et al., 2016; Creery et al., 2015; Schmidt et al., 2006), they found that a 2 h nap selectively benefitted weaker memories. However, the selective benefit for weaker memories diminished after a full night of sleep. A possible interpretation of these results is that weaker memories are reactivated earlier during sleep, i.e., are prioritized as they are more prone to forgetting. A full night of sleep, however, provides sufficient time to reactivate both weaker and stronger memories. While tempting, this interpretation requires additional research systematically controlling nap versus full night of sleep and weaker versus stronger memories. Another factor to be considered is the particular definition of weaker and stronger memories. For example, Tucker and Fishbein (2008) used a similar retrieval versus restudy manipulation as Bäuml et al. (2014) but came to different conclusions. They found a sleep-dependent consolidation effect for items subjected to retrieval practice (thought to result in stronger memories, see introduction), but not for items restudied (thought to result in weaker memories), which is the exact opposite pattern as in Bäuml et al. (2014). However, in their retrieval practice condition, Bäuml et al. (2014) had participants retrieve fewer items more frequently compared to Tucker and Fishbein (2008), likely to result in stronger memories. This illustrates the difficulty of categorically designating a particular memory as weak or strong based on behavioural assays alone. Real-time brain imaging might be used as a complementary measure to assess post-learning memory strength (Ezzyat et al., 2018).

## 5 Conclusion

Our study corroborates the beneficial effect of post-learning sleep for the consolidation of relatively weak memories. Critically though, we also show a sleep-dependent consolidation effect for relatively strong memories, which emerges when retrieval demands are increased to mitigate possible ceiling effects. These results suggest that all memories might undergo a consolidation process during sleep, raising the intriguing question whether and how hippocampal-cortical sleep dynamics differ for consolidating different types of memories.

## Author statement

**Marit Petzka**: Conceptualization, Methodology, Formal Analysis, Investigation, Data Curation, Writing – Original Draft, Visualization **Ian Charest**: Conceptualization, Software **George Balanos**: Resources, Supervision **Bernhard P. Staresina**: Conceptualization, Resources, Writing – Review & Editing, Supervision, Project administration, Funding acquisition.

## Funding

This work was supported by a 10.13039/100010269Wellcome Trust/10.13039/501100000288Royal Society Sir Henry Dale Fellowship to B.P.S. (107672/Z/15/Z).

## Data availability

Behavioural data, experimental paradigm and analysis code are publicly available on the Open Science Framework (https://osf.io/nf5r9/).

## Declaration of Competing Interest

None.

## Open practices

The study in this article earned Open Materials and Open Data badges for transparent practices.
