# Supplementary material for: Does sleep-dependent consolidation favour weak memories?
Source: Cortex. 2021 Jan;134:65–75. doi: 10.1016/j.cortex.2020.10.005 (PMC7805594; doi:10.1016/j.cortex.2020.10.005)
Supplement: Multimedia component 1 [file mmc1.docx]

# Supplemental Information. Does sleep-dependent consolidation favour weak memories?

Analogous to sequence consolidation, we conducted a 2x2x2 ANOVA for spatial consolidation with Delay (sleep vs. wake), Memory Strength (weaker vs. stronger) and Retrieval Difficulty (no interference vs. interference) as between-subjects factors. Spatial consolidation reflects the relative change in error rate (placement distance) from pre- to post-delay retrieval. Thus, values > 100% show an increase in error rate, < 100% show a decrease and values = 100% show a stabilization of error rate. Overall, post-relative to pre-delay error rate was significantly higher in wake groups than in sleep groups (main effect for Delay: F(1,112) = 9.22, p = .003, η_p_^2^= 0.08) and higher for high retrieval difficulty compared to low retrieval difficulty (main effect for Retrieval Difficulty: F(1,112) = 25.71, p < .001, η_p_^2^= 0.19). In contrast to sequence consolidation, we did not find a significant three way interaction (F(1, 112) = 0.34, p = .560, η_p_^2^< 0.01, BF_01_ = 4.57). Nevertheless, to fully characterise sleep-dependent consolidation effects for spatial memory, we conducted the same subsidiary ANOVAs as described in the main text.

First, a 2x2 ANOVA with the between-subjects factors Delay (sleep vs. wake) and Memory Strength (weaker vs. stronger) was used for the *no interference* groups only. Spatial consolidation did not significantly differ between sleep and wake groups (main effect Delay: F(1,56) = 2.59, p = .113, η_p_^2^= 0.04, BF_01_ = 1.28) and there was no modulation by initial memory strength (interaction of Delay x Strength: F(1,56) = 1.49, p = .228, η_p_^2^= 0.03, BF_01_ = 2.06, Figure S1A).


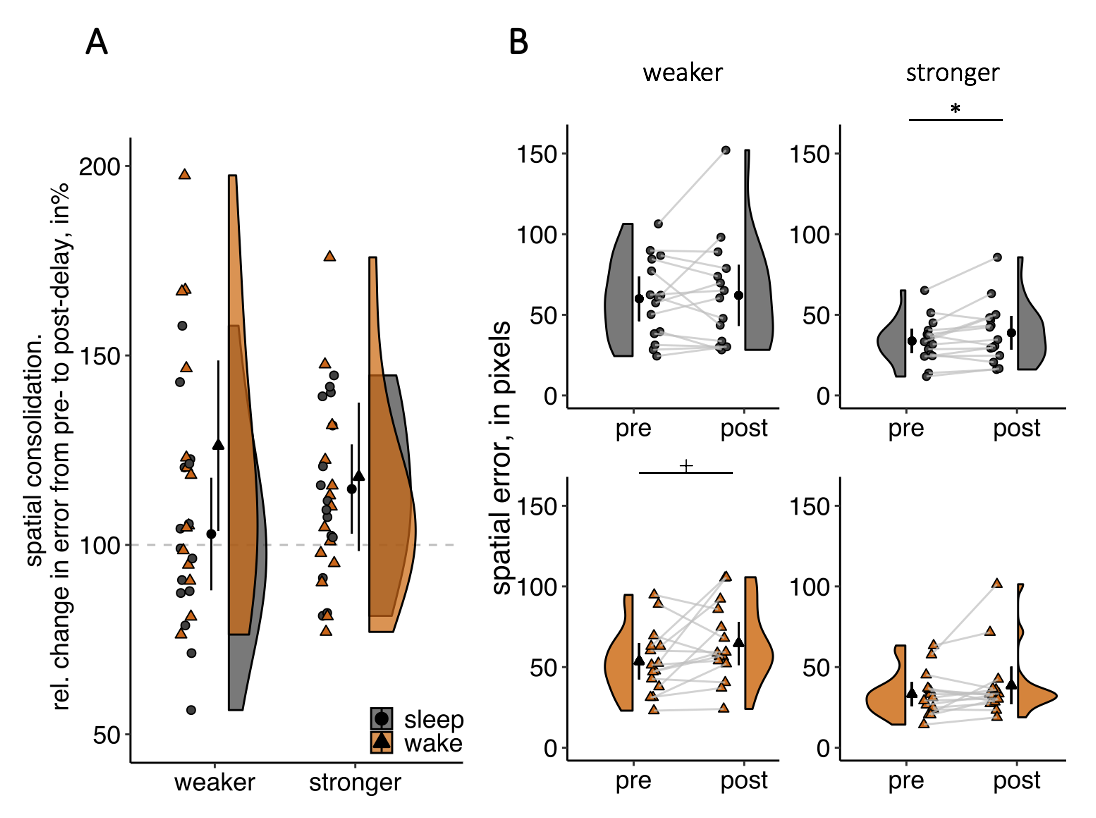


**Figure S1**. Spatial consolidation effects (error rate) for *no interference* groups. **A.** Neither for weaker nor for stronger memories did spatial consolidation (relative change in error rate from pre- to post-delay retrieval) significantly differ between the sleep (grey, circle) and the wake group (white, triangle). **B.** Pre- and post-delay error rate for weaker (*left column*) and stronger memories (*right column*).

Single participant data (grey filled circles for sleep groups and orange filled triangles for wake groups), density plots and group means with 95% CIs are shown in A and B. + = .1 ≥ p > .05; * = p ≤ .05

Second, a 2x2 ANOVA with the between-subjects factors Delay (sleep vs. wake) and Memory Strength (weaker vs. stronger) for the *interference* groups was conducted. In line with our notion that an increase in retrieval difficulty unveils sleep-dependent consolidation processes, we found a significant difference between the sleep and the wake groups (main effect Delay: F(1,56) = 7.68, p = .008, η_p_^2^= 0.12). Similar to sequence consolidation (see main text, section 3.3.), the sleep-dependent consolidation effect for spatial memory was comparable for weaker and stronger memories (no Delay x Strength interaction: F(1,56) = 0.12, p = .727, η_p_^2^< 0.01, BF_01_ = 3.64, Figure S2A), albeit only reaching significance for stronger memories (t(18.14) = -2.31, p = .033, d = 0.84) and not for weaker memories (t(15.33) = -1.64, p = .121, d = 0.60, BF_01_ = 1.07).


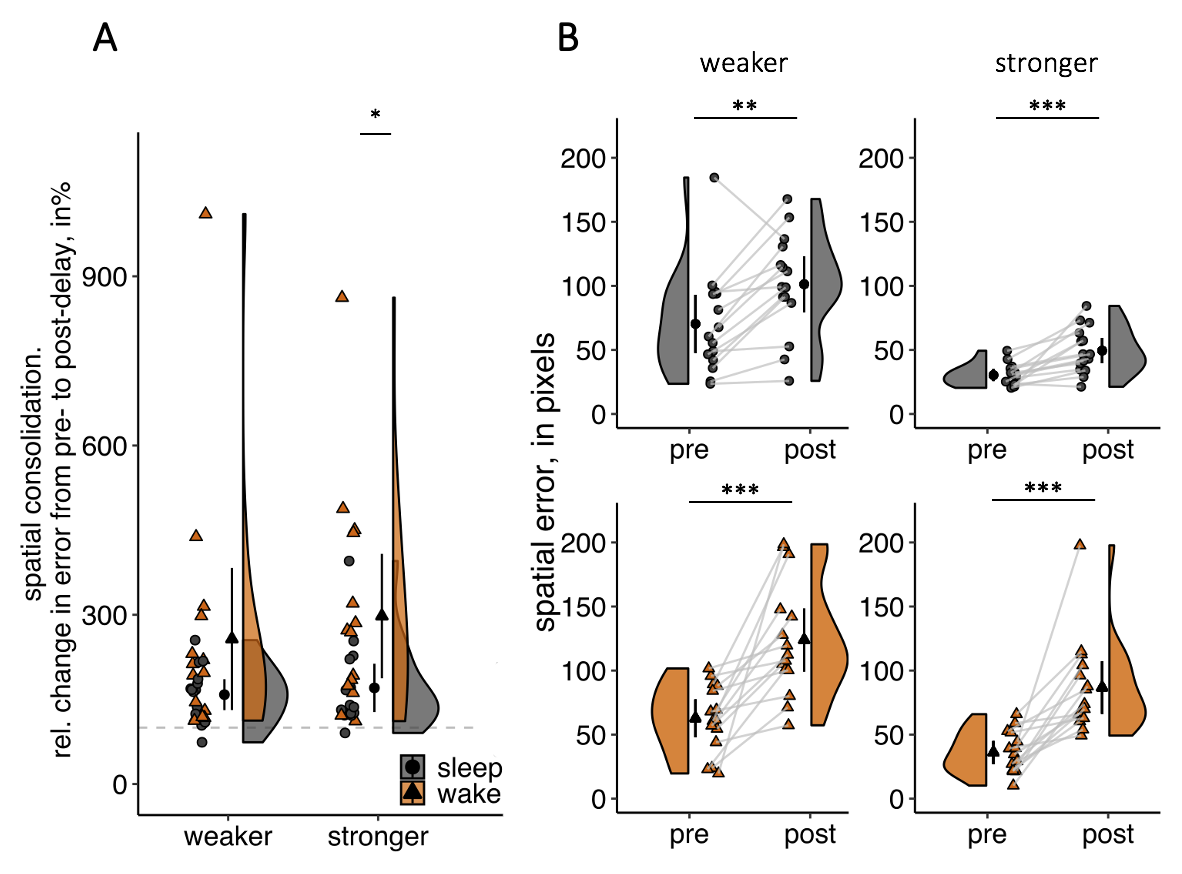


**Figure S2**. Spatial consolidation effects (error rate) for *interference groups*. **A.** Overall, spatial consolidation is significantly worse (greater relative change in error rate from pre- to post-delay retrieval) for the wake group (white, triangle) than for the sleep group (grey, circle). Note that results did not qualitatively change when outliers (N = 3) were excluded. **B.** Pre- and post-delay error rate for weaker (*left column*) and stronger memories (*right column*).

Single participant data (grey filled circles for sleep groups and orange filled triangles for wake groups), density plots and group means with 95% CIs are shown in A and B. * = p ≤ .05; ** = p < .01; *** = p < .001

Lastly, we conducted two 2x2 ANOVAs with the between-subjects factors Delay and Retrieval Difficulty for weaker and stronger memories, respectively. For stronger memories the sleep-dependent consolidation effect significantly increased from low to high retrieval difficulty (interaction Delay x Retrieval Difficulty for stronger memories: F(1,56) = 4.88, p = .031, η_p_^2^= 0.08). Note that an increase in sleep-dependent consolidation is reflected by more negative values as the error rate was used. For weaker memories, there was an overall trend for a sleep-dependent consolidation effect (main effect Delay, F(1,56) = 3.95, p = .0518, η_p_^2^= 0.07), without a significant interaction of Delay x Retrieval Difficulty (F(1,56) = 1.51, p = .225, η_p_^2^= 0.03, BF_01_ = 2.24, Figure S3).

**
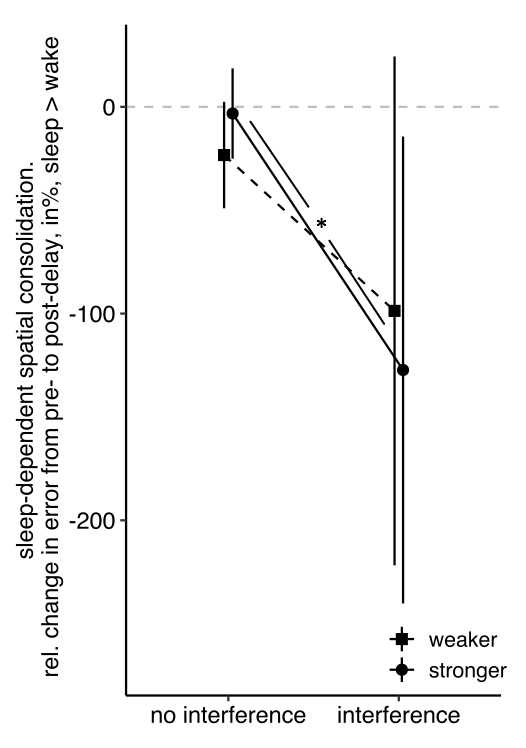
**

**Figure S3**. Sleep-dependent consolidation effects. For stronger memories, the difference in spatial consolidation (relative change in error rate from pre- to post-delay retrieval) between sleep and wake significantly increased with higher retrieval difficulty (interference). Shown are differences in means between sleep and wake groups and the corresponding 95% CIs. * = p ≤ .05

In sum, while increased retrieval difficulty also unveiled a sleep-dependent consolidation effect for strong spatial memories, effects for weak spatial memories were more subtle than for their sequence memory counterparts. One explanation might be that sequence memory was more sensitive to our initial memory strength manipulation (see main text, section 3.1. and 3.2.). Note also that both variables were operationalized on different scales. While sequence performance for an object is a binary outcome (correct or incorrect position in the sequence), spatial performance (i.e., spatial error) is a continuous variable (Euclidean distance from centre to centre in pixels). Further studies are needed in which temporal and spatial measures are more closely matched to adjudicate whether there are differential beneficial effects of sleep on temporal and spatial aspects of memory.

As our memory strength manipulation was defined based on the overall performance during training, we also used overall performance as dependent variable and conducted the same analyses as with sequence and spatial performance. Overall performance is the combined sequence and spatial performance, with higher values denoting better performance. Therefore, overall performance consolidation (relative change from pre- to post-delay) can be interpreted analogous to sequence consolidation: > 100% means an increase in performance, < 100% means a decrease and values = 100% mean stabilization of performance.

The results of the 2x2x2 ANOVA with Delay (sleep vs. wake), Memory Strength (weaker vs. stronger) and Retrieval Difficulty (no interference vs. interference) as between-subjects factors matched the results for sequence performance. We found two significant main effects for Delay (F(1,112) = 12.72, p < .001, η_p_^2^= 0.10) and Retrieval Difficulty (F(1,112) = 52.50, p < .001, η_p_^2^= 0.32). The three way interaction - despite only showing a trend towards significance (F(1, 112) = 3.09, p = .081, η_p_^2^= 0.03) - still suggests that sleep-dependent consolidation effects for weaker and stronger memories might differ as a function of retrieval difficulty. Therefore, we conducted the same subsidiary ANOVAs as described in the main text.

For the no interference groups, the 2x2 ANOVA with the between-subjects factors Delay (sleep vs. wake) and Memory Strength (weaker vs. stronger) showed almost the same pattern as in the main text. Overall performance consolidation was significantly greater in sleep compared to wake groups (main effect Delay: F(1,56) = 5.00, p = .029, η_p_^2^= 0.08) and this difference was modulated by the initial memory strength (interaction of Delay x Strength: F(1,56) = 5.39, p = .024, η_p_^2^= 0.09). Post hoc t-tests showed no significant difference in overall performance consolidation between sleep and wake group for stronger memories (t(28) = -0.08, p = .938, d = 0.03, BF_01_ = 2.90). However, for weaker memories, the sleep group showed significantly greater overall performance consolidation than the wake group (t(27.93) = 2.72, p = .011, d = 0.99, Figure S4A).


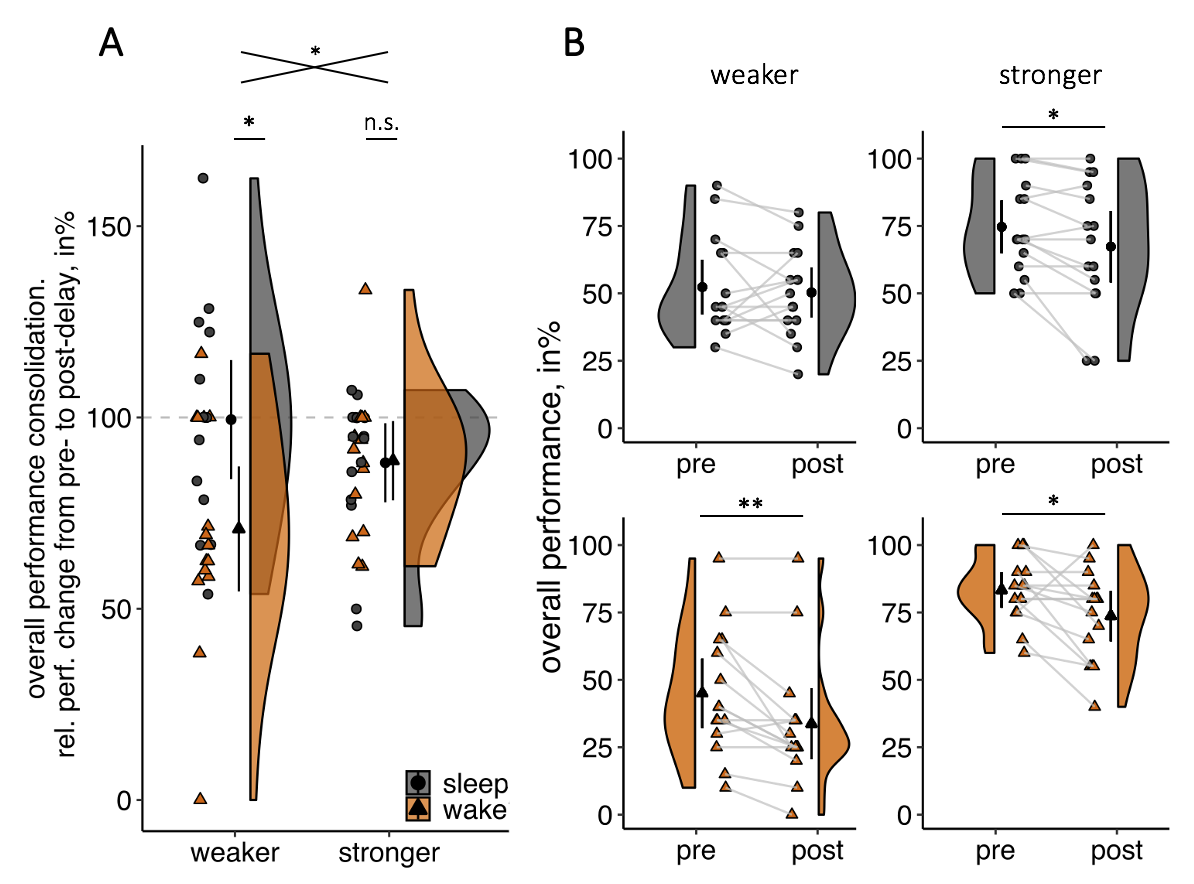


**Figure S4**. Consolidation effects for *no interference* groups. **A.** For weaker memories, overall performance consolidation (relative performance change from pre- to post-delay retrieval) is significantly greater in the sleep group (grey, circle) than in the wake group (orange, triangle), whereas there is no statistical difference between the sleep and wake group for stronger memories. **B.** Pre- and post-delay error rate for weaker (*left column*) and stronger memories (*right column*).

Single participant data (grey filled circles for sleep groups and orange filled triangles for wake groups), density plots and group means with 95% CIs are shown in A and B. * = p ≤ .05; ** = p < .01; n.s. = not significant, p > .1

After increasing retrieval difficulty by inducing retroactive interference, we still found a higher overall performance consolidation in the sleep groups than in the wake groups (main effect of Delay: F(1,56) = 7.73, p = .007, η_p_^2^= 0.12). Importantly, both weaker and stronger memories showed a significant sleep-dependent consolidation effect (no Delay x Strength interaction: F(1,56) = 0.17, p = .684, η_p_^2^= 0.003, BF_01_ = 3.57), indicating that both weaker and stronger memories benefited from post-learning sleep (Figure S5A).

**
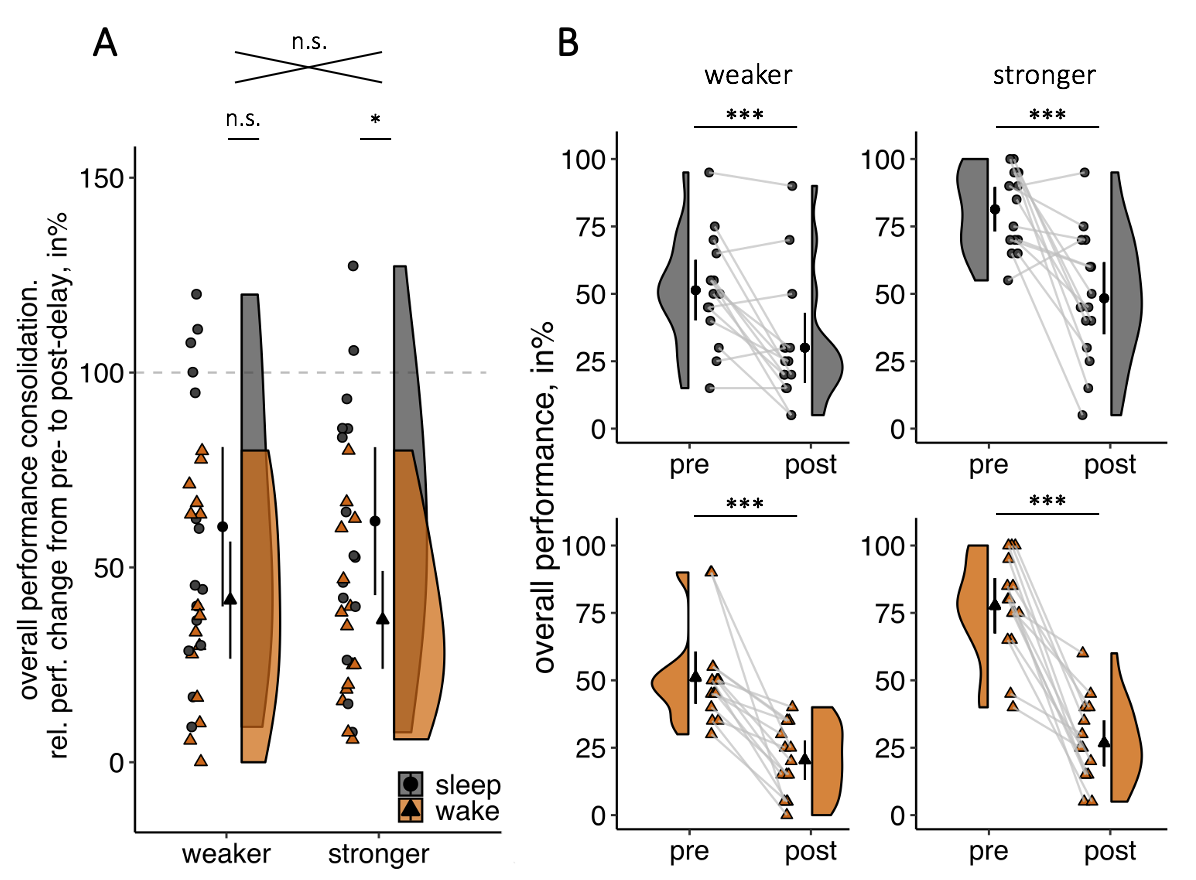
**

**Figure S5**. Consolidation effects for *interference* groups. **A.** After inducing retroactive interference, overall performance consolidation (relative performance change from pre- to post-delay retrieval) is significantly greater in the sleep group (grey, circle) than in the wake group (orange, triangle) for stronger memories. **B.** Pre- and post-delay error rate for weaker (*left column*) and stronger memories (*right column*).

Single participant data (grey filled circles for sleep groups and orange filled triangles for wake groups), density plots and group means with 95% CIs are shown in A and B. * = p ≤ .05; ** = p < .01; n.s. = not significant, p > .1

In a last step, we conducted two 2x2 ANOVAs with the between-subjects factors Delay and Retrieval Difficulty for weaker as well as for stronger memories. For weaker memories, the increase in retrieval difficulty had no impact on sleep-dependent consolidation effects (no interaction Delay x Retrieval Difficulty for weaker memories: F(1,56) = 0.38, p = .541, η_p_^2^= 0.01, BF_01_ = 3.41). For stronger memories, sleep-dependent consolidation effects significantly increased from low to high retrieval difficulty (interaction of Delay x Retrieval Difficulty: F(1,56) = 4.21, p = .045, η_p_^2^= 0.07, Figure S6).

Taken together, the results using overall performance largely correspond to the results using sequence performance as dependent variable.


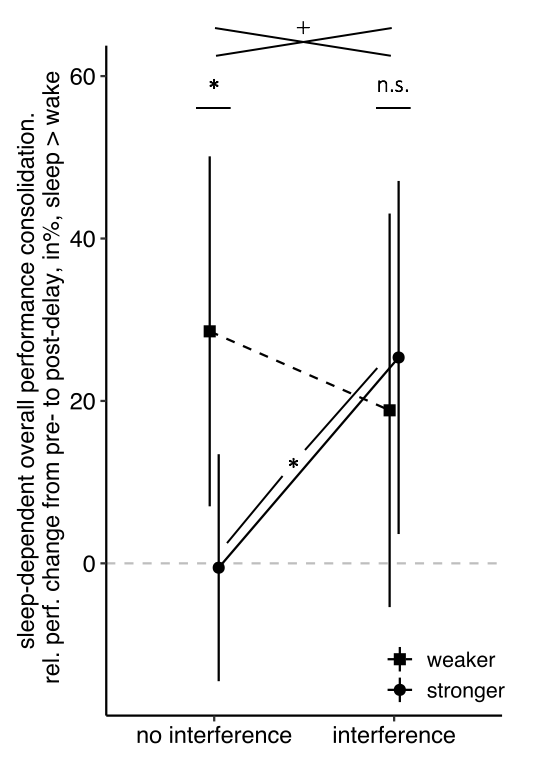


**Figure S6**. Sleep-dependent consolidation effects, overall performance. With low retrieval difficulty (no interference), the difference in overall performance consolidation (relative performance change from pre- to post-delay retrieval) between sleep and wake is significant for weaker memories only. With an increase in retrieval difficulty (interference), sleep-dependent consolidation effects are seen for both weaker and stronger memories. Shown are differences in means between sleep and wake groups and the corresponding 95% CIs. + = .1 ≥ p > .05; * = p ≤ .05; n.s. = not significant, p > .1

**Table S1**. Descriptive data of duration (in seconds, encoding + training) and number of training rounds needed to reach the criterion. Mean ± 95% CIs

|  | Weaker | | | | Stronger | | | | |
| --- | --- | --- | --- | --- | --- | --- | --- | --- | --- |
|  | **No interference** | | **Interference** | | **No interference** | | **Interference** | | |
|  | **Sleep** | **Wake** | **Sleep** | **Wake** | **Sleep** | **Wake** | **Sleep** | **Wake** |  |
| Duration | 909.06± 240.27 | 866.87± 177.41 | 996.15± 391.47 | 867.31± 188.68 | 1065.82± 130.52 | 1256.70± 208.00 | 1079.53± 187.81 | 1110.46± 248.21 |  |
| Rounds | 3.07± 1.65 | 3.27± 1.30 | 3.40± 1.85 | 3.13± 0.98 | 4.33± 0.58 | 5.47± 1.22 | 4.40± 0.95 | 5.33± 1.27 |  |

**Table S2**. Descriptive data of spatial memory performance (placement distance) pre and post- delay and the corresponding dependent t-tests. Mean ± 95% CIs, t-values, p-values. Significant effects are highlighted in bold.

|  | No interference | | | | | | Interference | | | | | |
| --- | --- | --- | --- | --- | --- | --- | --- | --- | --- | --- | --- | --- |
|  | **Weaker** | | | **Stronger** | | | **Weaker** | | | **Stronger** | | |
|  | **Sleep** | **Wake** | **Sleep** | | **Wake** | **Sleep** | | **Wake** | **Sleep** | | **Wake** |  |
| Pre-delay | 59.98± 13.85 | 53.58± 11.28 | 33.77± 7.62 | | 33.21± 7.51 | 70.34± 22.60 | | 62.70± 14.71 | 30.31± 4.60 | | 36.02± 8.94 |  |
| Post-delay | 62.03± 18.84 | 64.68± 13.34 | 38.80± 10.35 | | 38.57± 11.63 | 101.29± 21.79 | | 123.85± 24.43 | 49.46± 9.88 | | 86.73± 20.52 |  |
| t-value  (p-value) | -0.41  (.69) | -2.01  (.06) | **-2.34**  **(.03)** | | -1.59  (.13) | **-3.80**  **(<.01)** | | **-4.84**  **(<.01)** | **-4.33**  **(<.01)** | | **-5.19**  **(<.01)** |  |

**Table S3**. Descriptive data of overall memory performance pre- and post- delay and the corresponding dependent t-tests. Mean ± 95% CIs, t-values, p-values. Significant effects are highlighted in bold.

|  | No interference | | | | | | Interference | | | | | |
| --- | --- | --- | --- | --- | --- | --- | --- | --- | --- | --- | --- | --- |
|  | **Weaker** | | | **Stronger** | | | **Weaker** | | | **Stronger** | | |
|  | **Sleep** | **Wake** | **Sleep** | | **Wake** | **Sleep** | | **Wake** | **Sleep** | | **Wake** |  |
| Pre-delay | 52.33± 10.14 | 45.00± 12.86 | 74.67±  9.79 | | 83.33± 6.59 | 51.33± 11.20 | | 51.00± 9.66 | 81.33±  8.21 | | 77.66± 10.19 |  |
| Post-delay | 50.33± 9.15 | 33.67± 13.13 | 67.33± 13.23 | | 73.67±  9.33 | 30.00± 12.95 | | 20.33± 7.21 | 48.33± 13.35 | | 26.67± 8.48 |  |
| t-value  (p-value) | 0.57  (.58) | **3.65**  **(<.01)** | **2.75**  **(.02)** | | **2.45**  **(.03)** | **4.16**  **(<.01)** | | **5.65**  **(<.01)** | **4.33**  **(<.01)** | | **8.56**  **(<.01)** |  |

**Table S4**. Descriptive data of sequence memory performance pre- and post- delay and the corresponding dependent t-tests. Mean ± 95% CIs, t-values, p-values. Significant effects are highlighted in bold.

|  | No interference | | | | | | Interference | | | | | |
| --- | --- | --- | --- | --- | --- | --- | --- | --- | --- | --- | --- | --- |
|  | **Weaker** | | | **Stronger** | | | **Weaker** | | | **Stronger** | | |
|  | **Sleep** | **Wake** | **Sleep** | | **Wake** | **Sleep** | | **Wake** | **Sleep** | | **wake** |  |
| Pre-delay | 70.52± 13.53 | 62.11± 12.18 | 87.37± 7.85 | | 95.78± 4.00 | 61.75± 14.88 | | 65.26± 8.66 | 92.63±  7.29 | | 95.09± 4.04 |  |
| Post-delay | 68.42± 12.41 | 45.26± 14.14 | 85.97± 7.61 | | 88.77± 7.54 | 45.26± 14.06 | | 31.93± 10.06 | 72.63± 9.30 | | 42.11± 11.55 |  |
| t-value  (p-value) | 0.58 (.57) | **4.45**  **(<.01)** | 0.81  (.43) | | 1.66  (.12) | **2.94**  **(.01)** | | **5.51**  **(<.01)** | **3.99**  **(<.01)** | | **10.79**  **(<.01)** |  |

**Table S5**. Test statistic (W) and corresponding p values of the Shapiro-Wilk Test for pre- and post-delay sequence performance. Significant effects are highlighted in bold.

|  | Weaker | | | | | Stronger | | | | | | | |
| --- | --- | --- | --- | --- | --- | --- | --- | --- | --- | --- | --- | --- | --- |
|  | **No interference** | | | **Interference** | | | | **No interference** | | | **Interference** | | |
|  | **Sleep** | **Wake** | **Sleep** | | **Wake** | | **Sleep** | | **Wake** | **Sleep** | | **Wake** |  |
| Pre-delay | 0.92 (.23) | 0.95  (.51) | 0.90  (.10) | | 0.95  (.58) | | **0.80**  **(<.01)** | | **0.56**  **(<.01)** | **0.62**  **(<.01)** | | **0.63**  **(<.01)** |  |
| Post-delay | 0.92  (.23) | 0.94  (.43) | 0.91  (.12) | | 0.95  (.55) | | **0.85**  **(.02)** | | **0.78**  **(<.01)** | 0.96  (.72) | | 0.98  (.98) |  |

**Table S6**. Test statistic (W) and corresponding p values of the Shapiro-Wilk Test for pre- and post-delay spatial performance. Significant effects are highlighted in bold.

|  | Weaker | | | | | Stronger | | | | | | | |
| --- | --- | --- | --- | --- | --- | --- | --- | --- | --- | --- | --- | --- | --- |
|  | **No interference** | | | **Interference** | | | | **No interference** | | | **Interference** | | |
|  | **Sleep** | **Wake** | **Sleep** | | **Wake** | | **Sleep** | | **Wake** | **Sleep** | | **Wake** |  |
| Pre-delay | 0.96 (.62) | 0.95  (.59) | **0.86**  **(.03)** | | 0.94  (.36) | | 0.97  (.82) | | 0.92  (.17) | 0.92  (.22) | | 0.96  (.67) |  |
| Post-delay | **0.87**  **(.03)** | 0.95  (.51) | 0.97  (.84) | | 0.93  (.24) | | 0.92  (.18) | | **0.68**  **(<.01)** | 0.97  (.84) | | **0.80**  **(<.01)** |  |

**Table S7**. Test statistic (W) and corresponding p values of the Shapiro-Wilk Test for pre- and post-delay overall performance. Significant effects are highlighted in bold.

|  | Weaker | | | | | Stronger | | | | | | | |
| --- | --- | --- | --- | --- | --- | --- | --- | --- | --- | --- | --- | --- | --- |
|  | **No interference** | | | **Interference** | | | | **No interference** | | | **Interference** | | |
|  | **Sleep** | **Wake** | **Sleep** | | **Wake** | | **Sleep** | | **Wake** | **Sleep** | | **Wake** |  |
| Pre-delay | **0.87 (.03)** | 0.96  (.67) | 0.98  (.96) | | **0.80**  **(<.01)** | | 0.91  (.15) | | 0.94  (.42) | 0.90  (.11) | | 0.92  (.17) |  |
| Post-delay | 0.98  (.99) | **0.83**  **(.01)** | **0.82**  **(<.01)** | | 0.93  (.27) | | 0.94  (.35) | | 0.96  (.61) | 0.99  (.99) | | 0.96  (.65) |  |
